# Impact of Carbonyl Group Incorporation in Semicrystalline High-Density Polyethylene

**DOI:** 10.1021/acs.macromol.5c02288

**Published:** 2025-10-23

**Authors:** Afiq Anuar, Arman Edalat, Lea Ringelhan, Qiang Yu, Maximilian Baur, Albrecht Petzold, Stefan Mecking, Thomas Thurn-Albrecht, Kay Saalwächter

**Affiliations:** † Institut für Physik, Martin-Luther-Universität Halle-Wittenberg, 06099 Halle (Saale), Germany; ‡ Department of Chemistry, University of Konstanz, 78457 Konstanz, Germany

## Abstract

Ketone-functionalization of polyethylene via copolymerization with carbon monoxide offers a promising route to introducing reactive carbonyl moieties while preserving the advantageous bulk properties of high-density polyethylene (HDPE). Here, we systematically investigate the influence of low-level (0.6–1.6 mol %) keto incorporation on the thermal properties, semicrystalline morphology, crystallization, and chain dynamics of HDPE. Differential scanning calorimetry and small-angle X-ray scattering reveal only minor reductions in melting temperature and lamellar thickness. Complementarily, ^1^H NMR FID measurements reveal that KetoPE samples exhibit crystallinity-temperature profiles comparable to HDPE, indicating that the semicrystalline morphology is mainly preserved upon keto incorporation up to a few percent. Importantly, ^13^C *T*_1_ relaxation quantitatively confirms that intracrystalline chain diffusion coefficients are essentially unchanged. Notably, ^1^H spin-diffusion NMR confirms that presumably the isolated carbonyl moieties predominantly reside in the interphase. Thus, low-level ketone incorporation imparts additional reactivity or adhesion potential without compromising HDPE’s mechanical or thermal integrity.

## Graphical abstract

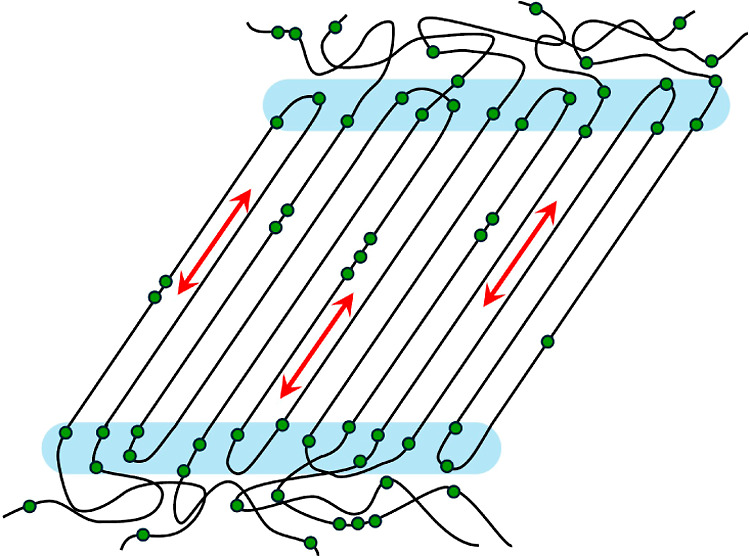

## 1 Introduction

High-density polyethylene (HDPE) remains a cornerstone in the polymer industry due to its exceptional mechanical strength, chemical resistance, and thermal stability. These properties have led to its widespread use in packaging and other applications where durability and cost-effectiveness are critical. However, HDPE’s high crystallinity and hydrophobicity limit its degradability, contributing to environmental concerns and restricting its range of applications. To address these limitations, considerable effort has been devoted to modifying HDPE via comonomer incorporation or chemical functionalization, aiming to introduce novel reactive sites while preserving its desirable bulk properties.^1–7^

Among these strategies, incorporating small amounts of functional groups such as carbonyl (keto) groups into the polyethylene backbone has long been explored as a means to enhance photodegradability and improve compatibility with polar surfaces.^8–12^ Early approaches, such as oxidation or milling, enabled keto incorporation but often resulted in heterogeneous and poorly controlled distributions of carbonyl groups.^8,9^ Recently, advances in catalytic copolymerization have allowed the precise placement of keto moieties, either regular or randomly distributed, at defined concentrations, revitalizing interest in ketone-functionalized polyethylene (KetoPE) as a versatile material platform.^4,10–14^ Additionally, these KetoPE materials are also able to form dynamic cross-linked networks via imine chemistry,^15^ which may improve recyclability and processing, thereby broadening the potential applications of sustainable polyethylene-based materials. In particular, some of us have recently demonstrated that low levels of precisely^4^ or randomly^10^ incorporated keto groups (up to a few mol %) in KetoPE produced materials with thermal and mechanical properties nearly indistinguishable from HDPE, suggesting that this approach may offer a practical path to functionalized, degradable polyethylene.

Despite these promising initial observations, the effects of low-level keto group incorporation on the thermal properties, semicrystalline morphology, and crystalline structure of HDPE deserve closer attention. Especially, the influence of keto groups on intracrystalline dynamics, referring to chain mobility within the crystalline domains and their exchange with amorphous regions, has not yet been thoroughly investigated. Such molecular motions underlie the mechanical α_c_-relaxation process observed in semicrystalline polyethylene and are reported to affect rheological behavior, tensile strength, thermal stability and, most importantly, the solid-state drawability.^16–19^ Despite their relevance, studies of intracrystalline dynamics in functionalized systems such as KetoPE remain limited, as probing these motions requires complex and specialized characterization techniques, a limitation that also constrains investigations in other polymer systems.

While prior work on ester-functionalized polyolefins suggests that irregular polar group placement can enhance molecular mobility by disrupting crystal packing,^20^ whether such effects occur in KetoPE remains unclear. Notably, Boyd and Sayre^9^ observed a dielectric relaxation process in slightly oxidized polyethylene containing approximately 0.1 mol % carbonyl groups, which was absent in unoxidized HDPE, suggesting that even low levels of oxidation-induced defects can facilitate molecular motion within the crystalline phase. We recently reported only minor changes in thermal and mechanical properties at higher keto incorporation levels of up to 1.0 mol %,^10^ suggesting little to no impact on intracrystalline dynamics. This discrepancy highlights the need for a more detailed understanding of how keto groups influence HDPE properties, particularly intracrystalline dynamics, which are governed by the crystalline structure and play a key role in determining the morphology and overall performance of semicrystalline polyethylene.

In this work, we systematically investigate the effects of small amounts of randomly distributed carbonyl groups, incorporated at concentrations ranging from 0.6 to 1.6 mol %, on the thermal properties, semicrystalline morphology, crystalline structure, and intracrystalline dynamics of KetoPE, synthesized via nickel-catalyzed nonalternating ethylene/carbon monoxide copolymerization.^10^ A combination of advanced characterization techniques was employed, including differential scanning calorimetry (DSC), small angle and wide-angle X-ray scattering (SAXS and WAXS), and temperature-dependent nuclear magnetic resonance (NMR) spectroscopy, to thoroughly investigate these properties. Our findings show that low-level keto incorporation leads to only minor to negligible changes in the thermal properties, the semicrystalline morphology and the intracrystalline chain diffusion, and thus in the properties of polyethylene. Solid-state ^13^C NMR experiments demonstrate that the keto groups can be accommodated within the crystalline regions of PE, revealing a practical route toward enhancing reactivity within the amorphous phase and interfacial adhesion while preserving the overall properties of semicrystalline HDPE.

Although the keto groups can be incorporated into the crystalline lamellae, our temperature-dependent ^1^H FID and ^13^C NMR analyses indicate that they neither promote nor hinder intercrystalline chain dynamics (ICD), specifically, the chain-sliding diffusion within the crystalline domains, explaining how KetoPE retains its original HDPE properties. Comprehensive ^13^C *T*_1_-relaxation and ^1^H spin diffusion experiments further suggest that a fraction of the keto groups, possibly the isolated ones, predominantly localize in the interphase region, aligning with earlier observations by Menges et al.,^21^ who reported preferential localization of ester carbonyl groups at the crystalline–amorphous interface in long-chain aliphatic polyesters. Overall, our findings provide a deeper understanding of structure–property relationships in functionalized polyolefins. These insights will aid in molecular design strategies aimed at developing high-performance, degradable polyethylene materials with improved environmental compatibility.

## 2 Experimental Section

### 2.1 Materials

In this study, high-density polyethylene (HDPE) and keto-functionalized polyethylene (KetoPE) were employed, for key parameters see Table 1. HDPE (mPE M5510 EP) was obtained from Lumicene, TotalEnergies (France), while KetoPE samples (0.6–1.6 mol % carbonyl content, determined by ATR–IR^10^) were synthesized at the University of Konstanz via a nonalternating ethylene/CO copolymerization using a nickel-based catalyst, enabling precise control over keto group incorporation.^10^ Throughout this manuscript, the samples are denoted as HDPE-*x* and K*y*-*x* (for KetoPE), where *M*_w_ ≈ *x* kg mol^–1^ and *y* mol % is the carbonyl content. For instance, K1.6-400 denotes a KetoPE sample with 1.6 mol % carbonyl content and *M*_w_ ≈ 400 kg mol^–1^. Most KetoPE samples feature ^13^C-labeled carbonyls (using labeled ^13^CO in the synthesis), facilitating solid-state NMR detection. The unlabeled K1.6-400 sample serves as a reference. The keto moieties in these samples appear as isolated carbonyl (IC), in double carbonyls separated by *n* ethylene repeat units (DC*n*) or in alternating polyketone (APK) motifs, which appear in a roughly 1:1:1 ratio on average, with a trend toward more ICs at low keto contents.^10^

**Table 1:** Molecular Characteristics of the Polyethylene (PE) Samples Used in This Study

sample	*M*_w_ (kg/mol)^a^	PDI^b^	label
HDPE-77	77.6	2.8	HDPE-77
^13^C KetoPE-0.6 mol %	396	1.8	K0.6-396
^13^C KetoPE-1.0 mol %	221	1.7	K1.0-221
^13^C KetoPE-1.5 mol %	90	1.5	K1.5-90
^12^C KetoPE-1.6 mol %	400	2.5	K1.6-400

a Weight-average molecular weight (in kg/mol).

b Polydispersity index.

### 2.2 ^1^H Low-Resolution NMR FID Analysis

^1^H time-domain NMR experiments were conducted on a 200 MHz Bruker AVANCE III Spectrometer, using a static probe head with a short dead time of 2 μs. The temperature was controlled during the experiment by heated or cooled air flow, operated using a BVT300 unit with an accuracy of ±1 K and a gradient up to 0.5 K over the sample. Stepwise heating was applied to realize the *T*-dependence in Figure 2a, with an additional 10 min of equilibriation time before each NMR-FID measurement. The pulse lengths and pulse power applied for 90° pulse were set to ≈2 μs (70–80 W). The recycle delay (RD) was set between 4 and 10 s, approximately 5 times the ^1^H *T*_1_ relaxation time of the crystalline domain, to ensure complete ^1^H magnetization relaxations of the sample.

The crystallinity of the samples is determined on a mobility basis, distinguishing between a crystalline phase with fast and nonexponential transverse relaxation arising from the strong ^1^H–^1^H dipole–dipole couplings in combination with only restricted dynamics, and more mobile components with motion-averaged dipolar couplings and thus slower transverse relaxation. The amplitudes of fast-, intermediate- and slow-decaying FID components reflect the crystal, interface and amorphous fractions, respectively. The fitting equation based on this concept reads^22^

1 $$I_{FID}\left(t\right)=A_{c}\cdot e^{-\left(a^{2}t^{2}/2\right)}\cdot \frac{\sin\left(b\cdot t\right)}{b\cdot t}+A_{i}\cdot e^{-{\left(t/T_{2,i}^{*}\right)}^{\upsilon_{i}}}+A_{a}\cdot e^{-{\left(t/T_{2,a}^{*}\right)}^{\upsilon_{a}}}$$

where *t* is acquisition time, *A*_c,i,a_ are the amplitude of the corresponding decaying component, *T*_2,i,a_^*^ and υ_a,i_ are the shape parameters apparent *T*_2_ and stretching exponents υ of the more mobile components, while *a* and *b* are the shape parameters of the crystalline part, where the so-called Abragamian function works well for polymers with only CH_2_ groups along the main chain. Figure 1 shows a representative example of FID curve fitting for HDPE-77 crystallized and measured at 125 °C. The NMR-based mass crystallinity was calculated according to

2 $$f_{c}=\frac{A_{c}}{A_{c}+A_{i}+A_{a}}$$

**Figure 1:**
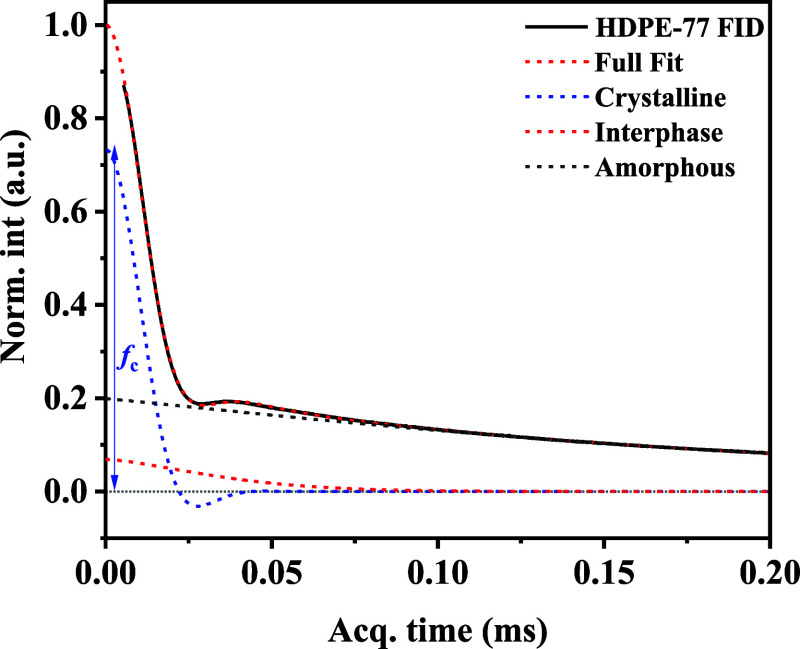
(a) Decomposition of the ^1^H FID NMR of HDPE measured at 125 °C.

### 2.3 ^13^C Magic-Angle Spinning NMR

All ^13^C MAS spectra measurements were conducted on 400 MHz Bruker AVANCE III and NEO spectrometers with a ^13^C Larmor frequency of 100.6 MHz using double- and triple-resonance magic-angle-spinning (MAS) probes at a rotation frequency of 10,000 ± 3 Hz. The sample temperature was approximately 115 °C in most cases (again realized via a heated airflow), sufficiently far below *T*_m_ but as high as possible to clearly observe possible effects of ICD. The π/2-pulse power used was set to 40 W for ^1^H and 140 W for ^13^C, with corresponding pulse lengths of approximately 3 μs each. ^13^C cross-polarization (CP) spectra were employed to enhance the weak ^13^C intensity. Due to the reduced efficiency of ^1^H–^13^C polarization transfer for mobile components, short contact times (CT) could be used to suppress the contribution from amorphous domains in the spectra (100 μs and about 1 ms for amorphous CH_2_ and carbonyl groups, respectively). ^13^C direct-polarization (DP) spectra with short recycle delay (RD) were used to selectively detect mobile groups within the sample. The RD was set to 1 s, ensuring that only signals with *T*_1_ relaxation shorter than 1 s, corresponding to mobile chains, were detected.

### 2.4 *T*_1_ Relaxation and Diffusive Exchange

The *T*_1_ relaxation behavior provides crucial insights into the molecular dynamics of ^13^C in the polymer chains. In this work, the ^13^C *T*_1_ relaxation measurements were performed using a *z*-filtered pulse sequence applied to the ^13^C channel after CP.^23^ This method ensures that the signal decay, represented by *I*_τ_, reaches a well-defined final value of zero intensity. The observed signal decay can reflect either exponential *T*_1_ relaxation or a diffusive process. The latter arises when intracrystalline chain diffusion transports the magnetization from crystalline regions to the amorphous phase, where *T*_1_ relaxation is almost instantaneous.^16^ A key signature of such diffusive behavior is a linear decay when the data is plotted as a function of the square root of time, $\sqrt{\tau }$ . To distinguish between diffusive and exponential relaxation, the decay function (1 – *I*_τ_/*I*_0_) can be analyzed on a double-logarithmic scale against τ.^19^ In this representation, a power-law signal rise with an exponent of 1/2 serves as a indicator of diffusive behavior, while a near-linear τ dependence corresponds to exponential relaxation in the short-time limit. This approach provides an effective method to differentiate and quantify contributions of chain diffusion and actual *T*_1_ relaxation.

### 2.5 ^1^H Spin Diffusion

The Goldman–Shen (GS) dipolar-filter experiment^24^ was performed to investigate crystal surface segments by using *T*_2,H_ filtering to suppress crystalline signal, and ^1^H spin diffusion to transfer ^1^H magnetization from the amorphous domain into the crystalline interphase, followed by CP to ^13^C. During the rotor-synchronized *T*_2,H_ filter, which had a duration around 0.4 ms (in multiples of the rotation period), the ^13^C–^1^H dipolar interaction was decoupled by irradiation on the ^13^C channel. The ^1^H spin diffusion time (after the *T*_2,H_ filter and before cross-polarization) ranged from 1 ms to 1 s. Afterward, CP from ^1^H to ^13^C was performed to observe the ^13^C signal.

### 2.6 Differential Scanning Calorimetry

Differential scanning calorimetry (DSC) was conducted using a DSC 8000 instrument (PerkinElmer). HDPE and KetoPE samples were heated to 180 °C for 10 min to remove thermal history, and then cooled to −60 °C at a rate of 10 K min^–1^. After being kept at −60 °C for 10 min, the samples were heated to 180 °C at a rate of 10 K min^–1^. Background contributions to the signal were subtracted, resulting in measurements of the apparent heat capacity *c*_p_(*T*).

### 2.7 Small-Angle and Wide-Angle X-ray Scattering

Small-angle X-ray scattering (SAXS) and wide-angle X-ray scattering (WAXS) measurements were carried out using a Retro-F laboratory setup (SAXSLAB, Copenhagen, Denmark) operated with a microfocus X-ray source combined with an ASTIX multilayer X-ray optics (AXO Dresden GmbH, Dresden, Germany) as a monochromator, yielding Cu Kα radiation with a wavelength (λ) of 0.154 nm. Two mm thick aluminum discs with a central hole were used as sample holders. The exposure time was 300 and 600 s for WAXS and SAXS measurements, respectively. The intensity of scattered X-rays was recorded by a 2D PILATUS3 R 300 K detector (DECTRIS Ltd., Baden, Switzerland) at different sample-to-detector distance (SAXS: 1035.5 mm, WAXS: 85.5 mm). An azimuthal average of the intensity collected with the 2D detector gives the scattering signal as a function of scattering vector *q* or *s* (*q* = 4π/λ sin θ = 2π*s*). *q* is the scattering vector used in the comparison of WAXS data, and *s* is the scattering vector used in SAXS. For temperature control, a hot stage with a TMS 94 temperature controller (Linkam, United Kingdom) was used.

All SAXS measurements were quantitatively analyzed based on the interface distribution function,^25,26^ providing the average crystalline (*d*_c_) and amorphous layer thicknesses (*d*_a_). With these parameters, the linear crystallinity can be determined as *f*_c,SAXS_ = *d*_c_/(*d*_c_ + *d*_a_). The details of the evaluation of SAXS data can be found in our previous publications.^25,26^ A detailed analysis on one of our samples is described in the Supporting Information, Figure S1. A scattering contribution attributed to partial aggregation of the keto groups, best visible in melt-state data shown in Figure S2, that partially survived also in the semicrystalline state, was subtracted.

## 3 Results and Discussion

### 3.1 Thermal Properties of Keto-Containing Polyethylene

Differential scanning calorimetry (DSC) was used to examine the impact of keto incorporation on the thermal properties of polyethylene. Table 2 summarizes the melting temperatures (*T*_m_), melting enthalpies (Δ*H*_m_), and crystallization temperatures (*T*_c_) for HDPE and KetoPE samples obtained from the first cooling and second heating scans after removal of thermal history at 180 °C, as shown in Figure S3. We report peak maxima for *T*_m_ and *T*_c_, while Δ*H*_m_ was calculated by integrating the area under the melting peak using a linear baseline extrapolated from the heat capacity in the molten state.

**Table 2:** Summary of DSC Results^a^

sample	*T*_m_ (°C)^b^	Δ*H*_m_ (J/g)^c^	*T*_c_ (°C)^d^
HDPE-77	138.71	196.23	118.65
K0.6-396	134.59	175.83	116.81
K1.0-221	132.27	169.73	116.96
K1.6-400	132.29	161.85	117.63

a See Figure S3 for the data.

b Melting temperature.

c Melting enthalpy.

d Crystallization temperature.

A slight decrease in *T*_m_ and Δ*H*_m_, along with an even smaller change in *T*_c_, is observed with increasing keto content. For example, comparing sample K0.6-396 and K1.6-400 reveals that a 1 mol % increase in keto content leads to a minor reduction of ∼2.3 °C in *T*_m_ and only a ∼0.8 °C variation in *T*_c_. Notably, these KetoPE samples possess higher molecular weights, which would typically be expected to increase *T*_m_ and *T*_c_ due to reduced chain mobility and fewer chain ends. The fact that slight decreases are still observed suggests that keto groups may have a minor disruptive effect on crystallization. However, these changes remain minor, indicating that the addition of up to 1.6 mol % keto groups has little overall impact on the thermal properties of polyethylene. This observation is consistent with the prior DSC observations,^10^ where negligible differences were found between a different HDPE sample (Lyondellbasell Purell GB 7250) and KetoPE samples containing 0.3 to 1.0 mol % keto content. We also note that Nozaki et al.^11,13,14^ also reported slightly reduced melting temperatures for nonrandom keto-functionalized polyethylene as compared to HDPE, consistent with our observations for randomly distributed keto groups.

The DSC results suggest that keto incorporation has a minimal effect on the thermal properties of polyethylene, in strong contrast to a recent systematic investigation of the effect of small amounts of noncrystallizable comonomers.^27^ Octene comonomers were shown to reside in a diffuse interphase and to lead to a reduction of *T*_m_ and Δ*H*_m_ by 15 °C and 50%, respectively, at a comparable content of 1.6 mol % comonomers. We next investigated whether the observed small thermal changes correspond to noticeable alterations in semicrystalline morphology, now focusing on conditions of isothermal crystallization for a most systematic assessment. To explore this, small- and wide-angle X-ray scattering (SAXS/WAXS) measurements were performed to evaluate potential variations in crystalline and amorphous layer thicknesses, as well as the crystal unit cell dimenions.

### 3.2 Semicrystalline Morphologies

SAXS measurements were conducted to examine the effect of randomly distributed keto groups up to 1.6 mol % on the semicrystalline morphology of polyethylene, focusing on the crystalline (*d*_c_) and amorphous (*d*_a_) layer thicknesses. Table 3 summarizes these parameters after 2 h of isothermal crystallization at 115 °C, with measurements taken at the same temperature, along with their long period, *L* = *d*_c_ + *d*_a_, and the corresponding SAXS-based crystallinity, *x*_c,SAXS_ = *d*_c_/*L* (as a volume-based crystallinity it is somewhat smaller than the NMR-based *f*_c_).

**Table 3:** Morphology Data From In Situ SAXS Measurements after 2 h of Isothermal Crystallization at 115 °C

sample	*d*_*c*_ (nm)^a^	*d*_*a*_ (nm)^b^	*L* (nm)^c^	*x*_c,SAXS_ ^d^
HDPE-77	24.24	11.13	35.37	0.6854
K0.6-396	21.91	14.54	36.45	0.6010
K1.0-221	19.37	14.70	34.07	0.5685
K1.6-400	20.04	18.70	38.74	0.5270

a Crystalline layer thickness.

b Amorphous layer thickness.

c Long period.

d SAXS-based linear crystallinity *x*_c,SAXS_ = *d*_c_/*L*.

The results in Table 3 show no major changes in semicrystalline morphology with increasing keto content. For example, comparing K0.6-396 and K1.6-400, which have comparable molecular weights but differ by 1% in keto content, reveal only minor changes in both *d*_c_ and *d*_a_ by approximately 2–4 nm, with the overall semicrystalline dimension *L* changing only by ∼2.3 nm. Although *x*_c,SAXS_ decreases by 7.4% in this comparison, the overall morphological differences remain moderate, indicating that this level of keto incorporation does not substantially affect the semicrystalline structure of polyethylene. Additionally, supplementary wide-angle X-ray scattering (WAXS) measurements shown in Figure S4 further support this conclusion by showing that the scattering peaks of all KetoPE samples overlap with those of HDPE, indicating that the incorporation of keto groups does not significantly modify the unit cell dimensions.

Notably, the larger *d*_c_ compared to *d*_a_ in all KetoPE samples is a typical characteristic of so-called crystal-mobile polymers, in which chain diffusion occurs between crystalline and amorphous layer.^18,19,25,28^ We again refer to results of our previous study,^27^ where similar amounts of noncrystallizable octene comonomers were shown to drastically alter the morphology of polyethylene toward the crystal-fixed regime, where *d*_a_ becomes larger than *d*_c_ and the mechanically detected α_c_ relaxation becomes undetectable. The comparable semicrystalline morphology between all given samples suggests that the randomly placed keto moieties also do not hinder chain-sliding diffusion between the crystalline and amorphous layers,^18,25^ a point that will be further investigated using NMR measurements. Overall, the SAXS data imply that small, randomly distributed keto groups (up to 1.6 mol %) have little to no effect on the fundamental semicrystalline architecture of polyethylene. These findings align with DSC results, which showed only a slight disruption in thermal properties, particularly crystallization, as reflected in minor changes in *T*_c_ with increasing keto incorporation.

As DSC and SAXS/WAXS results indicate that keto incorporation up to 1.6 mol % induces only minor if not negligible modifications of the thermal properties and the semicrystalline morphology, the small observed variations warrant further investigation. To address this, ^1^H NMR FID measurements were conducted to examine crystallinity as a function of temperature and time after isothermal crystallization, providing deeper insights into crystallization stability and crystallization kinetics in keto-functionalized polyethylene.

### 3.3 Crystallization Behavior: Temperature Dependence and Kinetics

The semicrystalline properties of the keto-containing samples were further examined by measuring crystallinity as a function of temperature via ^1^H NMR FID. Each sample was first molten and then isothermally crystallized for 2 days at its respective *T*_c_ (fixed at identical undercooling below the respective *T*_m_) to allow extended lamellar thickening, enhancing sensitivity to potential effects of keto incorporation on crystallization. HDPE-77 was included as a reference to illustrate the typical crystallinity-temperature behavior of polyethylene, which remains stable over a wide temperature range.^29,30^ Crystallinity was extracted using the multicomponent fitting approach described in the Experimental Section and illustrated in Figure 1, distinguishing crystalline, interphase, and amorphous components.

Figure 2a presents results obtained upon reheating the isothermally crystallized and then cooled samples. HDPE-77 exhibits the expected nearly constant crystallinity across the covered temperature range (25–115 °C), while the keto-containing samples generally follow a similar trend with minor variations. A slight decrease in crystallinity is observed with increasing temperature for keto-containing samples, particularly K0.6-396 and K1.6-400, whose crystallinity-temperature profiles overlap substantially. Since both samples have comparable *M*_w_ but differ in keto concentration by 1%, this overlap may indicate that the observed reduction in crystallinity with temperature, suggestive of weaker crystal stability, is primarily influenced by *M*_w_ rather than keto content.

**Figure 2:**
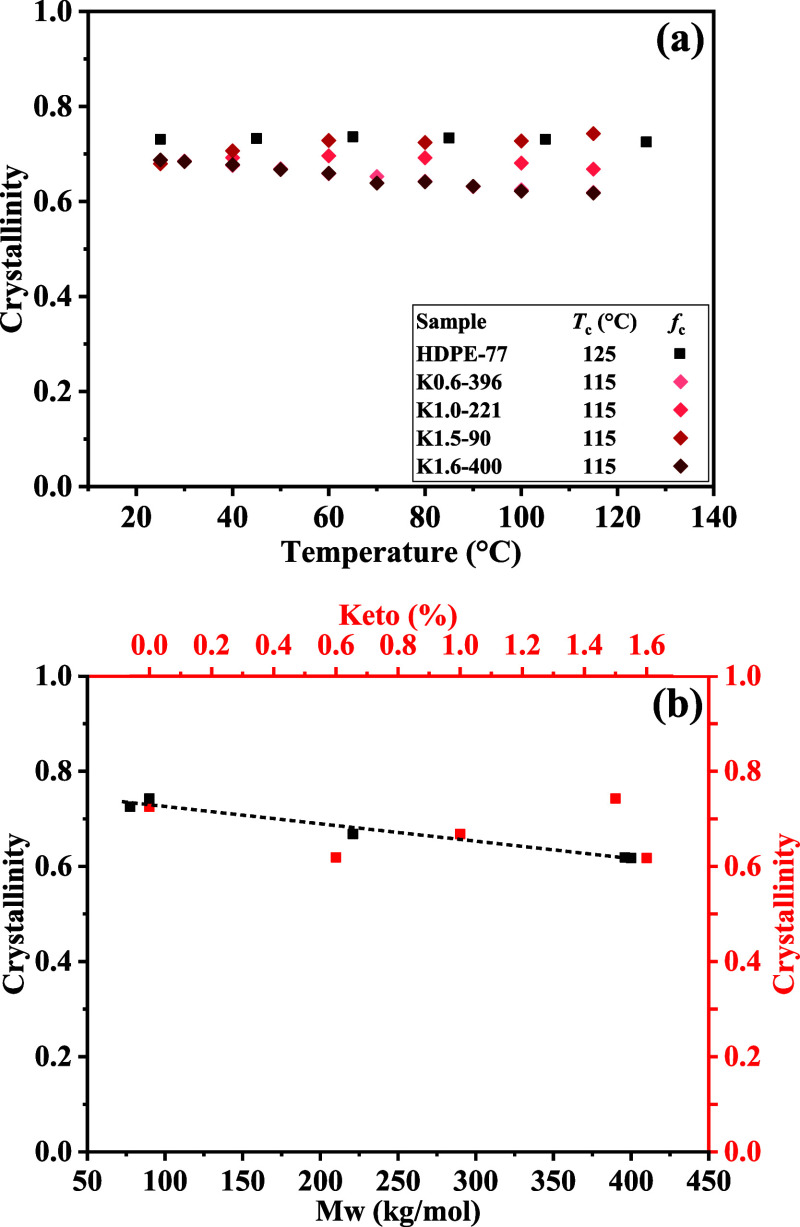
Crystallinity of HDPE and keto-containing samples obtained via ^1^H NMR FID. (a) Crystallinity as a function of temperature after isothermal crystallization for 2 days at the respective crystallization temperature *T*_c_ shown in the legend. HDPE-77 serves as a reference. (b) Crystallinity plotted against molecular weight (*M*_w_, left *y*-axis, black) and keto content (right *y*-axis, red), with values taken at the isothermal crystallization temperature *T*_c_ specified in (a), highlighting the stronger influence of *M*_w_ compared to keto concentration.

The dominant influence of *M*_w_ over keto inclusion is finally supported by Figure 2b, which shows crystallinity plotted against both *M*_w_ (black bottom axis) and keto content (red top axis), demonstrating the much clearer trend of crystallinity reduction with changes in *M*_w_, while the crystallinity fluctuates nonsystematically with respect to keto content. Additional time-resolved crystallization experiments (see Figure S5) further confirm that keto groups up to 1.6 mol % exert negligible influence on the crystallization behavior, as evidenced by comparable crystallization rates, preservation of prolonged secondary crystallization (which is typically sensitive to variations in intracrystalline chain dynamics, ICD), and a crystallinity trend governed primarily by molecular weight rather than keto content, fully consistent with the temperature-dependent crystallinity analysis.

Overall, the temperature-dependent crystallinity measurements, along with the crystallinity dependence shown in Figure 2b, with crystallinity values taken at the respective isothermal crystallization temperatures *T*_c_ (125 °C for HDPE-77 and 115 °C for the KetoPE samples), as well as the time-resolved crystallization in Figure S5 indicate that the incorporation of up to 1.6 mol % keto groups has only a minor influence on the thermal stability of semicrystalline polyethylene, consistent with the minimal changes observed in thermal properties and morphology from DSC and X-ray scattering analyses. Having established that the crystallinity characteristics remain largely unaffected by keto incorporation up to 1.6 mol %, despite their known dependence on intracrystalline dynamics, this observation appears to suggest that keto groups do not significantly hinder or promote ICD. To further explore this possibility, the following section examines the intracrystalline dynamics of KetoPE in detail using temperature-dependent ^1^H and ^13^C NMR measurements.

### 3.4 Intracrystalline Dynamics

Figure 3 presents the normalized crystalline ^1^H NMR FID shapes of all samples measured at different temperatures, based upon the same data set as Figure 2. The evolution of these lineshapes with temperature provides insights into the presence of ICD within the acquisition time scale.^19,28,31^ The results show that all samples exhibit comparable temperature-dependent changes. The lack of significant differences among the samples, which also confirms the presence of ICD, suggests that the keto incorporation up to 1.6 mol % does not substantially alter the PEs’ ICD time scales. This is further supported by the temperature-dependent behavior of the second moment of the dipolar line shape *M*_2_, shown in Figure S6, where all samples exhibit a comparable nonlinear decrease with temperature due to lattice expansion as well as motional narrowing.

**Figure 3:**
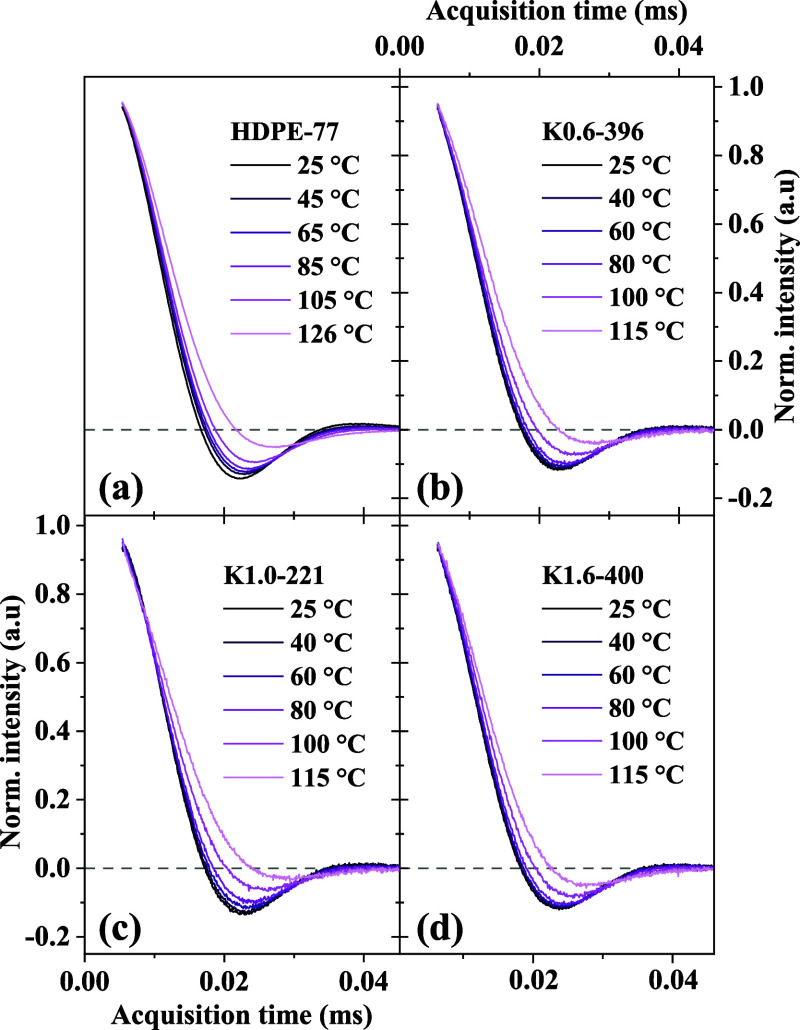
Normalized 1H NMR-FID crystalline lineshapes of (a) HDPE-77 and (b–d) keto-containing samples measured at different temperatures, illustrating the presence of intracrystalline dynamics (ICD). The progressive prolongation of the transverse relaxation decay (narrowing of the corresponding lineshapes after Fourier transformation) with increasing temperature indicates the presence of fast α_c_-relaxation processes. The similarity in line shape evolution across all samples suggests that keto incorporation has minimal influence on ICD time scales.

Although the normalized crystalline ^1^H NMR FID shapes suggested that the randomly distributed keto groups up to 1.6 mol % do not alter ICD, they do not provide a quantitative measure of chain diffusion between crystal and amorphous domains. To directly probe chain sliding and quantify the diffusivity, we employ ^13^C *T*_1_ relaxation measurements. Prior to these measurements, it is essential to determine which ^13^C resonances can be reliably attributed to the crystalline region.

Figure 4 presents the combined ^13^C MAS spectra of K1.5-90. As described in the Experimental Section, CP with a 1.5 ms contact time (black spectrum) enhances all ^13^C resonances, whereas CP with a 0.1 ms contact time (red spectrum) and DP spectra with 1 s recycle delay (blue spectrum) selectively accentuate crystalline and amorphous resonances, respectively. In addition, the resonance positions in these spectra distinguish signals from the crystalline and amorphous domains, leveraging the γ-gauche effect,^32,33^ where all-trans conformation typically exhibit slightly higher chemical shifts than gauche-containing segments. The selective ^13^C labeling on keto groups facilitates the detection of these dilute keto moieties, as highlighted by a comparison with a naturally abundant sample, see Figure S7.

**Figure 4:**
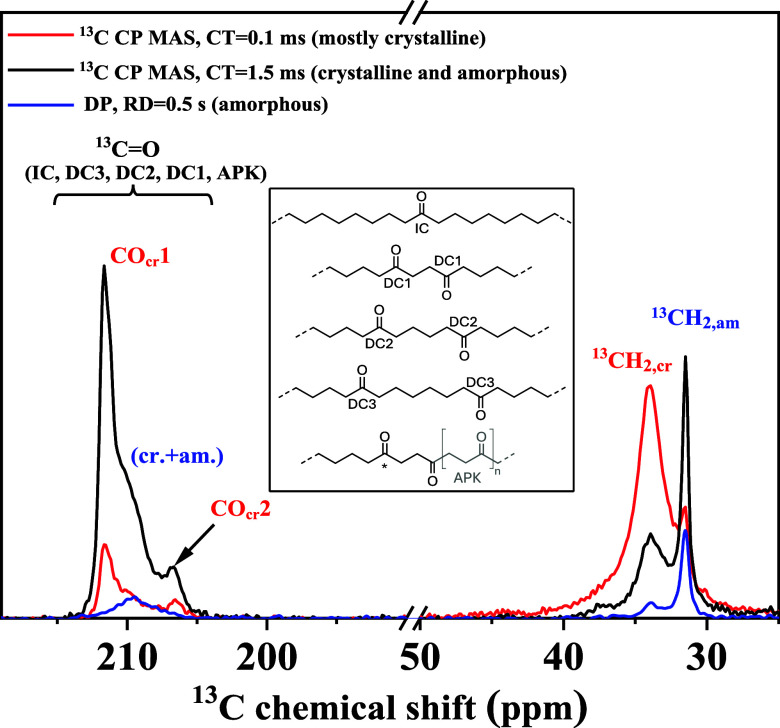
^13^C NMR spectra of ^13^C-labeled K1.5-90 measured at 10 kHz MAS and 115 °C. The red spectrum corresponds to ^13^C CP MAS with a contact time (CT) of 0.1 ms, the black spectrum to a CT of 1.5 ms and the blue one is a direct-polarization (DP) spectrum. Key resonances include ^13^CH_2_ (rigid, crystalline) at 30.2 ppm, ^13^CH_2_ (mobile) at 27.6 ppm, and rigid/crystalline ^13^CO at approximately 203-208 ppm. The inset illustrates the molecular structure and labeling scheme of keto-functionalized PE (KetoPE), indicating the carbonyl group types that could only be assigned in the solution state: IC, DC*n*, and APK (see the Materials subsection).

The spectra reveal methylene peaks that are typically observed in semicrystalline PE,^16,30,34^ along with additional carbonyl peaks from the dilute keto groups. The intensities of the methylene and carbonyl peaks are comparable in CP spectra with a 1.5 ms contact time, as the percentage of ^13^C in natural abundance is nearly equal to the percentage of ^13^C-labeled keto groups in the overall sample. The much weaker intensities of keto group peaks relative to methylene peaks in the DP spectra with 1 s recycle delay simply result from the longer ^13^C *T*_1_ relaxation time of carbonyls (confirmed in separate experiments), which are predominantly governed by chemical shift anisotropy rather than comparably stronger CH_2_ dipole–dipole interactions.^35^

Previously,^10^ the ^13^CO chemical shifts of ^13^C-keto-functionalized PE measured in solution could be assigned to different CO-ethylene comonomer sequences along the main chain as arising from the synthesis method, i.e., IC, DC*n*, and APK, across which the CO groups are distributed with an approximate relative ratio of 1:1:1 on average (see the Materials subsection). A detailed shift assignment in the given bulk (amorphous and crystalline) states is actually a nontrivial issue, as taken up further below. We restrict the discussion to two prominent carbonyl resonances associated with the crystals, CO_cr_1 and CO_cr_2 at ∼211.7 and ∼206.7 ppm, respectively. These are most distinct from the in-between range of chemical shifts where also the amorphous-phase carbonyls resonate. A deconvolution was used to extract the relative intensity of these resonances, see Figure S8, while a definite assignment and distinction of DC*n* and amorphous peaks was not possible.

The detection of crystal-related (i.e., at least dynamically highly constrained) carbonyl groups is noteworthy, given that our earlier DSC, SAXS, and ^1^H NMR FID analyses indicated minimal to negligible impact of the keto groups on crystallization, semicrystalline morphology, crystalline temperature stability, and crystallization kinetics. This observation suggests that small amounts of randomly distributed keto groups up to 1.6 mol % can integrate into the crystalline domain while preserving the overall properties of HDPE. Moreover, the successful incorporation of keto groups into the crystalline phase may be particularly significant for developing photodegradable polyethylene, as degradation in PE are typically hindered in crystalline regions due to their compact, well-ordered chain packing and higher resistance to oxidation.^36–40^

Having established that keto groups can be accommodated within the crystalline lattice without major structural disruption, we next investigate whether their presence influences chain diffusion within the crystalline domain. While the crystalline ^1^H NMR FID shape analysis suggested that keto incorporation does not alter the characteristic time scales of ICD, these measurements primarily confirm the presence of ICD, which could consist in back-and-forth jumps rather than directly probing chain exchange between crystalline and amorphous domains, i.e., quantitative assessment of diffusivity. To address this, we employ ^13^C *T*_1_ relaxation measurements, which enable direct and quantitative insight into chain sliding within the crystalline domain of keto-functionalized polyethylene.

Figure 5a presents the ^13^C *T*_1_ relaxation measurements obtained using CP with a short CT of 0.1 ms, which as mentioned earlier, predominantly excites the crystalline phase. 115 °C (not far from melting) was chosen as a suitable experimental temperature to observe possible chain diffusion as clearly as possible. As described in the Experimental Section, a characteristic feature of this process is a linear decay of intensity with respect to the square root of waiting time, $\sqrt{\tau }$ .^16,19^ The results shows that all samples exhibit this characteristic linear decay, confirming the presence of chain sliding within the crystalline domain. Furthermore, all samples seems to exhibit comparable initial slopes, suggesting a comparable chain diffusion rate, which will be quantified later. The crystal-related carbonyl groups are also included here, but will be discussed separately.

**Figure 5:**
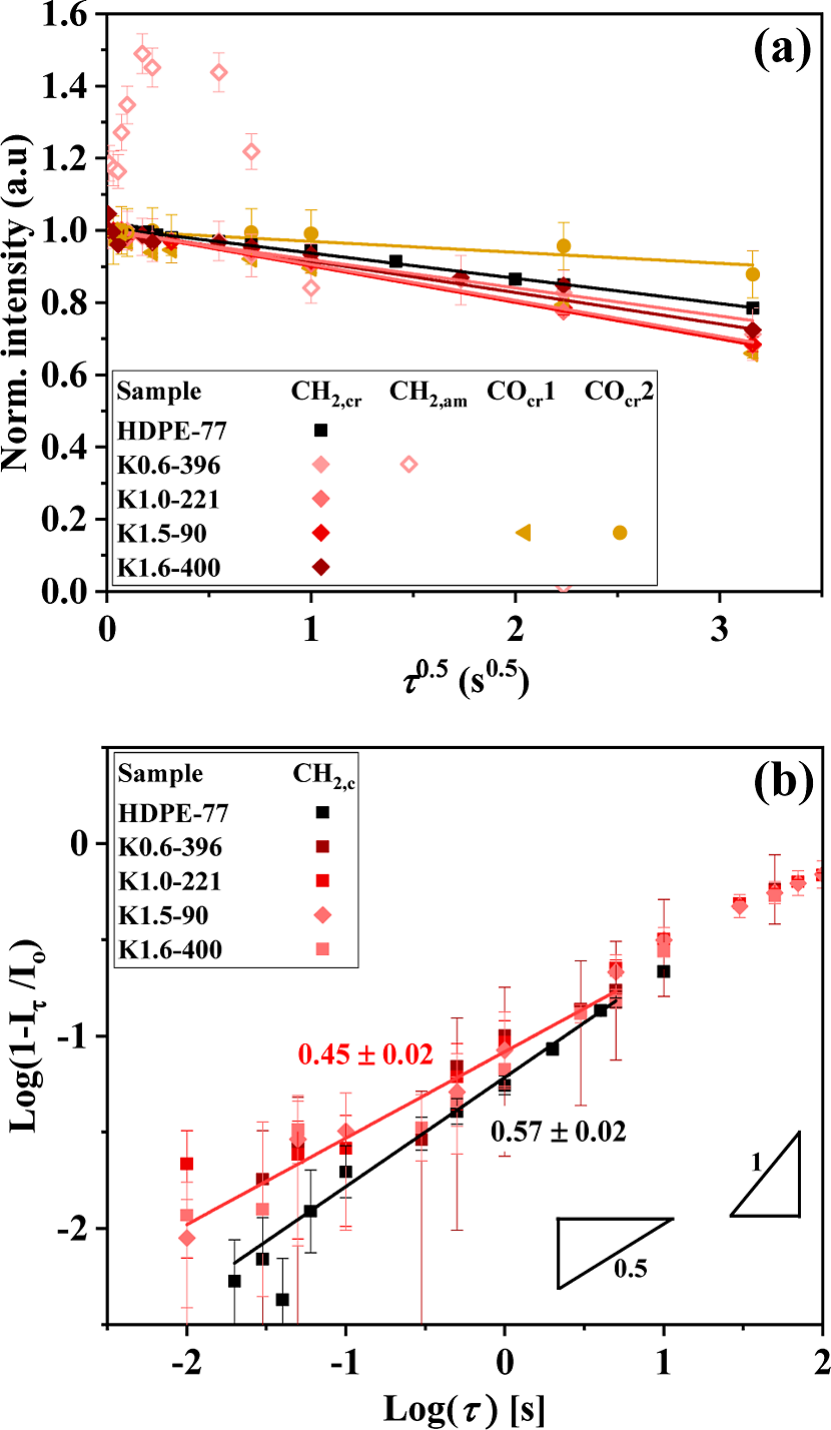
^13^C *T*_1_ relaxation experiments measured using CP with a CT of 0.1 ms at 115 °C. (a) Normalized intensity decay *I*_τ_/*I*_0_ for all samples. The crystalline CH_2,c_ signal decreases initially linearly with $\sqrt{\tau }$ , confirming the presence of chain diffusion. The initial magnetization buildup of the amorphous CH_2,a_ shown for one sample confirms the presence of chain exchange between crystalline and amorphous chains in all samples. (b) Double-logarithmic plot of (1 – *I*_τ_/*I*_0_) against τ, where the initial slopes indicate diffusive behavior of crystalline chains, with HDPE-77 exhibiting a slightly different slope compared to keto-containing samples.

Interestingly, the use of a short CT of 0.1 ms also revealed a buildup of intensity in the amorphous CH_2_ peak (open diamonds), before its comparably short *T*_1_ of ca. 1 s leads to ultimate signal loss. To ensure visual clarity, corresponding data for the other samples are presented separately in Figure S9. This intensity buildup originates from diffusion-mediated magnetization transfer from the highly magnetized crystalline phase, which is selectively polarized by the short CT, into the initially less magnetized amorphous domain, as further discussed in the context of Figure S9. To the best of our knowledge, this has not been reported in the literature as a method for detecting the presence of chain sliding into the amorphous phase. This observation also serves as direct evidence of chain diffusion also occurring into the amorphous region, aligning with other literature^16,34,41,42^ and further challenging previous studies that postulated α_c_-relaxations to be related exclusively to the crystalline phase.^43^

Returning back to the analysis of the crystalline CH_2_ peaks, the presence of chain sliding can also be further confirmed by a double-logarithmic plot of ^13^C *T*_1_-relaxation data, as demonstrated in our previous studies.^19^ In the case of diffusive behavior, the initial decay of the *T*_1_-relaxation intensity follows a 1D free-diffusion model, characterized by a square-root dependence on time, $\sqrt{\tau }$ ,

3 $$\frac{I_{\tau }}{I_{0}}=1-\frac{\sqrt{2D}}{d_{c}}\sqrt{\tau }$$

where *I*(τ)/*I*(0) represents the normalized intensity decay, *D* is the diffusion coefficient along the chain direction, and *d*_c_ is the crystalline thickness obtained from SAXS. In the absence of diffusion, the relaxation follows a simple exponential decay model.^16,19,30^ For further clarity, the 1D free-diffusion equation can be rewritten in logarithmic form

4 $$\log\left(1-\frac{I_{\tau }}{I_{0}}\right)=\frac{1}{2}\log\tau +\log\frac{\sqrt{2D}}{d_{c}}$$

This transformation provides the basis for a log–log plot of log­(1 – *I*_τ_/*I*_0_) vs log­(τ), where an initial slope of approximately 1/2 directly confirms power-law exponent characteristic of diffusive behavior. Deviations from this slope would suggest that the relaxation dynamics do not strictly follow the 1D diffusion model discussed here, which would be the case if actual *T*_1_ relaxation within the crystal would occur on a comparable time scale.

Figure 5b presents the double-logarithmic plot of (1 – *I*_τ_/*I*_0_) against τ for CH_2,c_ peak of all samples, which all samples exhibit an initial slope close to 1/2, providing a clearer *T*_1_-relaxation measurement feature that emphasizes the diffusive nature of the crystal chains. The KetoPE samples exhibit nearly identical initial slopes, with only a minor deviation observed for HDPE-77, consistent with the comparable slopes observed in Figure 5a. The minimal variation in initial slopes between all samples observed in Figure 5a,b suggests that all samples exhibit comparable chain-diffusion rates. Using the initial slope from Figure 5a and the crystalline thickness (*d*_c_) determined from SAXS (see Table 3), the chain diffusion coefficients (*D*) were calculated using eq 3 and are compiled in Table S1. The estimated *D* values range from appproximately ∼1.22 to 1.91 nm^2^/s, indicating that the incorporation of up to 1.6 mol % randomly distributed keto groups has a negligible effect on chain-diffusion behavior. These findings are in agreement with earlier studies on different PE morphologies^30^ and are further consistent with the results from DSC, SAXS, and ^1^H NMR analyses, confirming again that keto incorporation does not significantly alter the semicrystalline structure and dynamics of polyethylene.

### 3.5 Keto Groups

Finally, we take a closer look at the behavior and localization of the sparse keto groups, nicely enabled by the ^13^C labeling, which was initially conceived for the solution-NMR characterization, i.e., carbonyl content, clustering and shift assignment,^10^ the latter realized by multidimensional techniques. We benefit from these samples and now probe the local environment and mobility of carbonyl sites with ^13^C *T*_1_ measurements, but need to check more closely the chemical-shift assignment, which turns out to be nontrivial.

Focusing on the K1.5-90 sample, Figure 6a shows a slow-MAS melt-state spectrum of the carbonyl region, including as inset a saturation recovery measurement indicating slightly multiexponential *T*_1_ relaxation and an average *T*_1_ of around 5 s. The fully relaxed melt-state spectrum is compared with the solution-NMR spectrum from ref 10 taken in deuterated tetrachloroethane at elevated temperature. We note that this sample exhibits a slightly higher APK content as most other samples. Notably, the shift dispersion is completely different, precluding any assignment of the melt-state spectrum, which shows a wide dispersion and only one prominent peak at around 208 ppm. We also note that the APK peak in tetrachloroethane solution appears at about 207 ppm, while custom-synthesized pure APK has a carbonyl resonance at about 212 ppm when measured in a mixture of hexafluoroisopropanol and benzene.^10^ This reveals large solvent-related effects, either arising directly from shielding of the local packing environment or indirectly from different conformational averaging,^33^ induced by solvent related changes of conformer energies. Also, the clustering of the CO groups in the melt as evidenced by SAXS (see Figure S2) could well play a role here.

**Figure 6:**
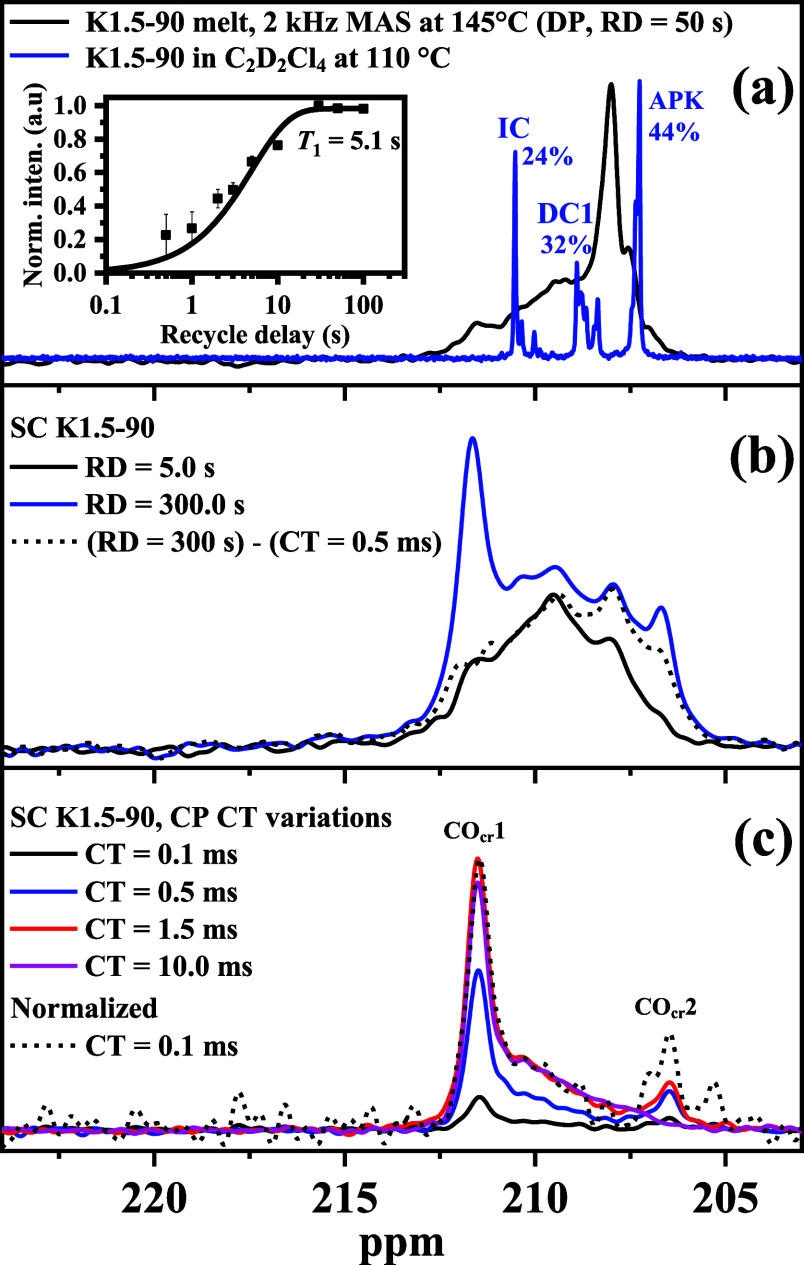
^13^C spectra (carbonyl region) of the ^13^CO-labeled KetoPE-1.5 mol % sample measured under different conditions: (a) melt-state DP spectrum at slow MAS (2 kHz) compared to a solution spectrum from ref 10 inset: saturation recovery curve; (b,c) semicrystalline-state (SC) spectra (b) with different recycle delay, including a relaxed spectrum after subtraction of the crystalline-only CP spectrum and (c) CP spectra with variable contact time.

In Figure 6b we compare a fully relaxed DP spectrum of the same sample in the semicrystalline state with an amorphous-only spectrum with short RD, where the measurement temperature of 115 °C is not too far from the molten state at 145 °C, meaning that thermal averaging over the conformers and thus the shift dispersion should be similar. After subtracting a suitably waited crystalline-only CP spectrum with the prominent CO_cr_1 and CO_cr_2 resonances, the amorphous-only spectrum is roughly reproduced, but it lacks the prominent peak at 208 ppm from the melt-state spectrum in panel (a). On this basis, we hypothesize that this peak may governed by ICs, which may have a preference to be related to the crystal and appear there as CO_cr_1. For completeness, variable-CT CP spectra are shown in Figure 6c, demonstrating a rather uniform build-up. Scaling up the shortest-CT spectrum to the maximum amplitude of the spectrum with a CT of 1.5 ms, we only recognize a somewhat larger CO_cr_2 resonance, yet this should not be overinterpreted, considering the noise level. The amorphous range is never prominent here, owing to larger ^13^C–^1^H distances of the CO group, motional averaging, and possibly short *T*_1ρ_.

We turn to the *T*_1_ relaxation behavior of the CO groups. Unlike for CH_2_ groups, whose *T*_1_ relaxation is predominantly governed by strong dipole–dipole interactions (2 CH dipolar tensors per group), ^13^CO *T*_1_ relaxation occurs via the overall smaller chemical shift anisotropy (CSA). This leads to significantly larger *T*_1_ values, in particular in the amorphous phase. Figure 7 presents the normalized *T*_1_ decays in ^13^C CP MAS spectra with a CT of 1.5 ms for the ^13^CO-labeled K1.5-90 sample in the carbonyl resonance region. The results show that CO_cr_2 groups exhibit a rather long apparent ^13^C *T*_1_ relaxation time ca. 110 s, consistent with the behavior expected for ^13^C residing in a rigid and well-ordered crystalline environment. In contrast, CO_cr_1 exhibit a significantly shorter *T*_1_ relaxation time of about 26 s, comparable to that of amorphous carbonyl groups (CO_am_). The longer *T*_1_ of CO_am_ compared to amorphous CH_2,am_ groups is expected, as the different groups are governed by interaction tensors of different magnitude. The surprising similarity in *T*_1_ values between CO_cr_1 and CO_am_ suggests the former population is considerably more dynamic and/or located in the crystalline–amorphous interphase. This statement is to be understood in terms of overall average residence time (the equilibrium population), as diffusion through the crystal should after all be possible.

**Figure 7:**
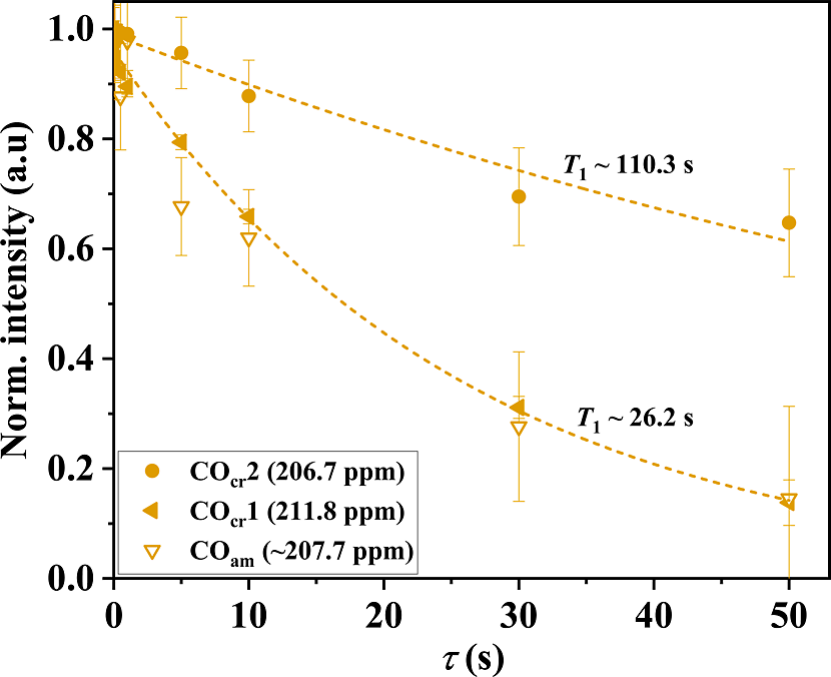
Normalized *T*_1_ intensity decay in ^13^C CP MAS spectra with a CT of 1.5 ms for the ^13^CO-labeled KetoPE-1.5 mol % sample measured at 115 °C.

The *T*_1_ decays of CO_cr_1 and CO_cr_2 are also included in Figure 5a, where we see that CO_cr_2 decays more slowly than CO_cr_1. The absolute decay rate cannot be easily interpreted in terms of chain diffusion, as the *T*_1_ in the amorphous phase (CO_am_) is a lower bound and not quasi-instantaneous, as is the case for the methylene groups. But the absolute-scale difference is again compatible with CO_cr_1 located closer to the interface than CO_cr_2. The shift difference may be due to different local chain conformations or also due to the comonomer sequence, i.e., IC vs DC*n* or APK.

To further examine the localization of keto groups within the HDPE crystalline region, ^13^C-detected ^1^H spin-diffusion experiments were performed at 5 kHz MAS using the Goldman–Shen (GS) dipolar-filter technique.^24^ This method selectively suppresses the crystalline signal, allowing ^1^H magnetization from the amorphous phase to diffuse into the crystalline surface over a controlled period via spin diffusion, as shown in Figure 8a. The plot illustrates that as spin diffusion time increases (on a time scale much shorter than chain-sliding diffusion!), the CH_2,cr_ peak intensifies while the CH_2,am_ peak diminishes, indicating magnetization transfer from the amorphous region into the crystalline surface. Interestingly, the CO_cr_1 peak exhibits a considerably faster magnetization buildup than CH_2,cr_, which is more clearly seen in Figure 8b. This proves that, indeed, these specific CO groups reside mostly in the interphase region.

**Figure 8:**
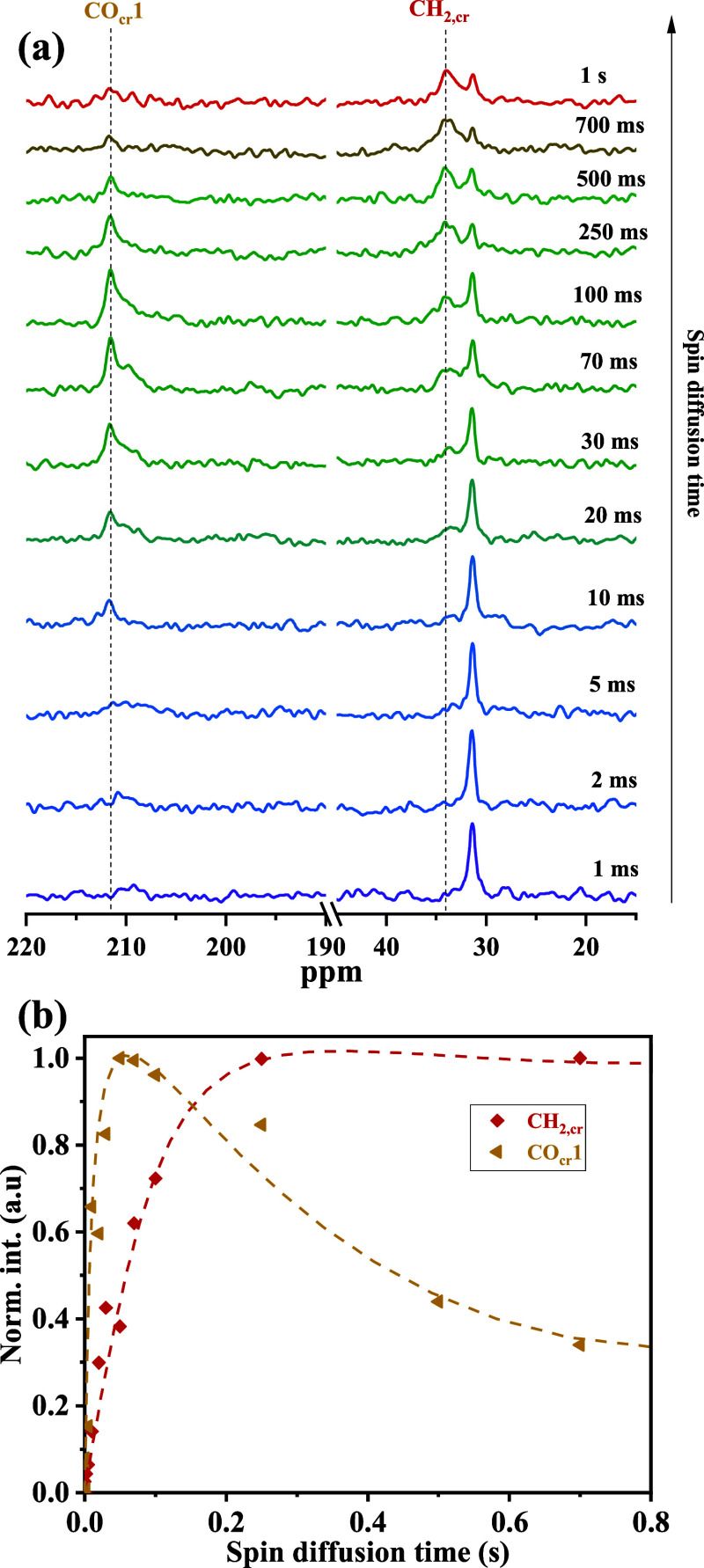
(a) ^13^C CP MAS spectra obtained from the Goldman–Shen (GS) dipolar filter experiment at 115 °C with variable ^1^H spin diffusion time. (b) Magnetization buildup for crystalline CO_cr_1 (red) and CH_2,cr_ (black) peaks. The faster buildup of CO_cr_1 magnetization suggests that a significant portion of these keto groups reside in the interphase region, supporting their role as structural defects leading to chain folding and localization at crystalline-adjacent regions.

These findings can be compared to a report by Menges et al.,^21^ where its was found that ester carbonyl groups remained predominantly near the crystal–amorphous interface in crystallized long-chain aliphatic polyesters. Menges et al.^21^ also reported that short-range flips or axial translations within the lamellae do not significantly displace the ester carbonyls from their original positions in that case. Instead, these substituents remain anchored at or near fold surfaces, where packing irregularities are more easily accommodated. Based on this notion, the increase in crystallinity over time observed in Figure S5, the existence of chain sliding as shown in Figures 3 and 5, as well as the predominant interphase localization of CO_cr_1 observed in Figures 7 and 8, are all consistent with a mechanism where lamellar thickening occurs predominantly with unsubstituted and possibly multiply substituted chain segments. A schematic of the semicrystalline structure of KetoPE, inspired by the model of Menges et al.,^21^ is shown in Figure 9, suggesting that most isolated CO groups are located in the interphase, while other groups of keto groups are more randomly distributed within the crystalline (as well as the amorphous) domain.

**Figure 9:**
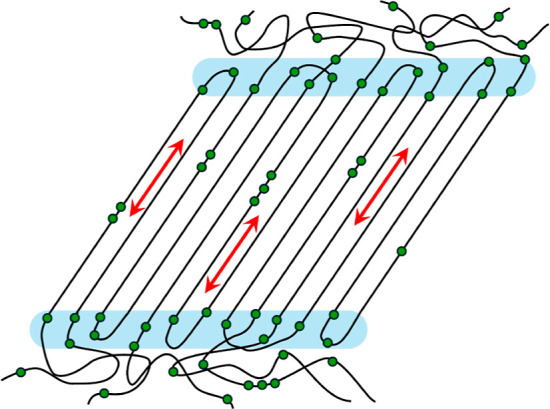
Schematic representation of the semicrystalline structure of KetoPE, illustrating the localization of randomly incorporated carbonyl (keto) groups (green circles). Most isolated carbonyls (IC_cr_) are hypothesized to be preferentially located near the crystalline–amorphous interface, indicated by the blue-shaded region, while a only smaller fraction as well as the clustered CO sites are also distributed within the crystalline lamellae.

## 4 Conclusions

This work demonstrated that the incorporation of up to 1.6 mol % of randomly distributed keto groups into high-density polyethylene (HDPE) exerts only minimal influence on its fundamental thermal properties, semicrystalline architecture, and chain dynamics. Differential scanning calorimetry (DSC) and small-angle X-ray scattering (SAXS) analyses revealed minor reductions in melting temperature and lamellar thickness, with negligible changes in overall crystallinity. This was further corroborated by ^1^H NMR FID results, confirming stable crystallinity over extended temperature ranges and also revealing similarly prolonged secondary crystallization kinetics, a hallmark of polymers exhibiting intracrystalline dynamics (ICD). These collective findings underscore that keto functionalization at these modest levels does not appreciably impair lamellar organization or crystal stability.

From a molecular-dynamics perspective, temperature-dependent ^1^H NMR line shape measurements and second-moment data confirmed that all keto-containing samples retain the same key feature as typical HDPE: readily detectable α_c_-relaxation within the crystalline domains. Furthermore, ^13^C *T*_1_ relaxation measurements performed on the crystalline methylene resonances unveil nearly unchanged diffusion coefficients, indicating that chain sliding within and out of the crystalline lattice proceeds at rates comparable to that of unmodified HDPE. These results emphasize the tolerance of semicrystalline polyethylene toward introducing carbonyl defects, as long as the total keto content remains at the percent level.

The ^13^C and ^1^H spin-diffusion NMR experiments suggested that a distinct carbonyl population (presumably the isolated ones, IC) appears to preferentially reside in the interphase region, similarly observed previously in semicrystalline long-chain aliphatic polyesters.^21^ We emphasize that this represents a time-averaged picture: even isolated carbonyls must be expected to also enter the crystal transiently during chain diffusion. Curiously, another smaller fraction (maybe paired (DC*n*) and alternating (APK) ones) does not have this preference and is thus distributed more evenly. These peculiarities do not seem to have any significant influence on the semicrystalline morphology as well as the bulk mechanical^10^ and thermal properties, although we acknowledge that further dedicated studies, particularly under nonlinear deformation conditions, would be worthwhile in future work. It may be mentioned that in a direct comparison between precisely placed ester vs precisely placed isolated carbonyl groups,^4^ only the latter showed virtually no influence of the functional-group content on the melting point of the materials, suggesting that chain-sliding diffusion is maintained also in that case. An NMR study of these materials would of course be highly worthwhile.

By preserving the desirable attributes of HDPE, namely its high crystallinity and mechanical robustness while adding sites for reactivity or adhesion, keto-functionalized polyethylene presents an attractive avenue for next-generation, eco-friendlier polyolefins. Potential applications include coatings, blends, and composites requiring improved interfacial bonding, as well as materials engineered for controlled degradation via photochemical or chemical routes that exploit the reactive carbonyl functionality. This strategy of targeted functional-group incorporation, alongside precise synthetic control, can guide the design of specialty polyolefins that bridge performance demands and sustainability goals, ultimately broadening the materials toolkit for advanced polymer applications.

## Supplementary Material



## Data Availability

The data sets generated and analyzed for this study as they appear in the figures of this article and the Supporting Information can be found in the Zenodo repository: 10.5281/zenodo.16736507.

## Notes

SAXS fitting procedure (Figure S1), melt-state SAXS data (Figure S2), DSC heating and cooling curves (Figure S3), WAXS diffractograms (Figure S4), crystallization kinetics from ^1^H NMR FID data (Figure S5), NMR dipolar second moments (*M*_2_) of all samples (Figure S6), ^13^C CP MAS NMR spectra of ^13^C-labeled and nonlabeled samples (Figure S7), ^13^C MAS NMR peak deconvolution of the carbonyl region (Figure S8), *T*_1_ data of amorphous CH_2,a_ (Figure S9), intracrystalline diffusion coefficients (*D*) and related data (Table S1) (PDF)

## References

[1] Boz E., Wagener K. B., Ghosal A., Fu R., Alamo R. G. (2006). Synthesis
and crystallization of precision admet polyolefins containing halogens. Macromolecules.

[2] Boz E., Nemeth A. J., Alamo R. G., Wagener K. B. (2007). Precision ethylene/vinyl
bromide polymers. Adv. Synth. Catal..

[3] Alamo R. G., Jeon K., Smith R., Boz E., Wagener K. B., Bockstaller M. (2008). Crystallization of polyethylenes containing chlorines:
Precise vs random placement. Macromolecules.

[4] Ortmann P., Wimmer F., Mecking S. (2015). Long-Spaced Polyketones from ADMET
Copolymerizations as Ideal Models for Ethylene/CO Copolymers. ACS Macro Lett..

[5] Santonja-Blasco, L. ; Zhang, X. ; Alamo, R. G. Crystallization of precision ethylene copolymers. In Polymer Crystallization I: From Chain Microstructure to Processing; Reiter, G. , Strobl, G. R. , Eds.; Springer, 2017; pp 133–182.

[6] Zhang X., Santonja-Blasco L., Wagener K. B., Boz E., Tasaki M., Tashiro K., Alamo R. G. (2017). Infrared spectroscopy and x-ray diffraction
characterization of dimorphic crystalline structures of polyethylenes
with halogens placed at equal distance along the backbone. J. Phys. Chem. B.

[7] Zhang X., Zhang W., Wagener K. B., Boz E., Alamo R. G. (2018). Effect
of self-poisoning on crystallization kinetics of dimorphic precision
polyethylenes with bromine. Macromolecules.

[8] Sayre J. A., Swanson S. R., Boyd R. H. (1978). The effect of pressure
on the volume
and the dielectric relaxation of linear polyethylene. J. Polym. Sci., Polym. Phys. Ed..

[9] Boyd R. H., Sayre J. A. (1979). Dielectric
evidence for thermal creation of defects
in polyethylene crystals. J. Polym. Sci. Polym.
Phys. Ed.

[10] Baur M., Lin F., Morgen T. O., Odenwald L., Mecking S. (2021). Polyethylene materials
with in-chain ketones from nonalternating catalytic copolymerization. Science.

[11] Tang S., Seidel F. W., Nozaki K. (2021). High Density
Polyethylenes Bearing
Isolated In-Chain Carbonyls. Angew. Chem., Int.
Ed..

[12] Voccia M., Odenwald L., Baur M., Lin F., Falivene L., Mecking S., Caporaso L. (2022). Mechanistic insights
into ni (ii)-catalyzed
nonalternating ethylene–carbon monoxide copolymerization. J. Am. Chem. Soc..

[13] Yuan H., Takahashi K., Nakagawa S., Yoshie N., Nozaki K. (2025). Linear Polyethylene
with Ketone Groups for Photodegradability: Higher Efficiency with
Side-Chain Carbonyls than In-Chain. ASC Macro
Lett..

[14] Nobis M., Takahashi K., Uchida J., Nakagawa S., Yoshie N., Kato T., Nozaki K. (2025). Polyethyleneketones with Controlled
Spacer Units: Synthesis, Characterization, and Photodegradation. J. Am. Chem. Soc..

[15] Morgen T. O., Mecking S. (2024). Circular Cross-Linked
Polyethylene Enabled by In-Chain
Ketones. ACS Macro Lett..

[16] Schmidt-Rohr K., Spiess H. (1991). Chain diffusion between crystalline and amorphous regions
in polyethylene detected by 2D exchange carbon-13 NMR. Macromolecules.

[17] Hu W.-G., Schmidt-Rohr K. (1999). Polymer ultradrawability:
the crucial role of α-relaxation
chain mobility in the crystallites. Acta Polym..

[18] Schulz M., Schäfer M., Saalwächter K., Thurn-Albrecht T. (2022). Competition
between crystal growth and intracrystalline chain diffusion determines
the lamellar thickness in semicrystalline polymers. Nat. Commun..

[19] Anuar A., Yu Q., Jariyavidyanont K., Petzold A., Androsch R., Thurn-Albrecht T., Saalwächter K. (2024). Poly-3-hydroxybutyrate, a crystal-mobile
biodegradable polyester. Macromolecules.

[20] Pepels M. P., Govaert L. E., Duchateau R. (2015). Influence
of the main-chain configuration
on the mechanical properties of linear aliphatic polyesters. Macromolecules.

[21] Menges M., Penelle J., Le Fevere
de Ten Hove C., Jonas A. M., Schmidt-Rohr K. (2007). Characterization
of long-chain aliphatic polyesters:
Crystalline and supramolecular structure of PE22, 4 elucidated by
x-ray scattering and nuclear magnetic resonance. Macromolecules.

[22] Schäler K., Roos M., Micke P., Golitsyn Y., Seidlitz A., Thurn-Albrecht T., Schneider H., Hempel G., Saalwächter K. (2015). Basic principles
of static proton low-resolution spin diffusion nmr in nanophase-separated
materials with mobility contrast. Solid State
Nucl. Magn. Reson..

[23] Torchia D. A. (1978). The measurement
of proton-enhanced carbon-13 T_1_ values by a method which
suppresses artifacts. J. Magn. Reson..

[24] Goldman M., Shen L. (1966). Spin-spin relaxation in LaF_3_. Phys.
Rev..

[25] Schulz M., Seidlitz A., Kurz R., Bärenwald R., Petzold A., Saalwächter K., Thurn-Albrecht T. (2018). The underestimated
effect of intracrystalline chain dynamics on the morphology and stability
of semicrystalline polymers. Macromolecules.

[26] Seidlitz, A. ; Thurn-Albrecht, T. Small-Angle x-ray Scattering for Morphological Analysis of Semicrystalline Polymers, Polymer Morphology: Principles, Characterization, and Prcessing; Wiley, 2016; p 153.

[27] Li S., Petzold A., Ranga A., Yu Q., van Niekerk M., Thurn-Albrecht T., Men Y. (2025). Effect of Noncrystallizable Comonomers
in Polyethylene on Crystallization, Semicrystalline Morphology, Intracrystalline
Dynamics, and Linear Mechanical Properties. Macromolecules.

[28] Yu Q., Anuar A., Petzold A., Balko J., Saalwächter K., Thurn-Albrecht T. (2023). The semicrystalline
morphology of polybutylene succinate
supports a general scheme based on intracrystalline dynamics. Macromol. Chem. Phys..

[29] Bärenwald R., Champouret Y., Saalwächter K., Schäler K. (2012). Determination
of chain flip rates in poly­(ethylene) crystallites by solid-state
low-field 1h nmr for two different sample morphologies. J. Phys. Chem. B.

[30] Bärenwald R., Goerlitz S., Godehardt R., Osichow A., Tong Q., Krumova M., Mecking S., Saalwächter K. (2014). Local flips
and chain motion in polyethylene crystallites: a comparison of melt-crystallized
samples, reactor powders, and nanocrystals. Macromolecules.

[31] Kurz R., Achilles A., Chen W., Schäfer M., Seidlitz A., Golitsyn Y., Kressler J., Paul W., Hempel G., Miyoshi T. (2017). Intracrystalline
jump
motion in poly­(ethylene oxide) lamellae of variable thickness: A comparison
of NMR methods. Macromolecules.

[32] Earl W. L., VanderHart D. (1979). Observations in solid polyethylenes
by carbon-13 nuclear
magnetic resonance with magic angle sample spinning. Macromolecules.

[33] Tonelli, A. E. NMR Spectroscopy and Polymer Microstructure: The Conformational Connection; VCH: Weinheim, 1989.

[34] Yao Y., Graf R., Spiess H. W., Lippits D., Rastogi S. (2007). Morphological
differences in semicrystalline polymers: Implications for local dynamics
and chain diffusion. Phys. Rev. E.

[35] Champmartin D., Rubini P. (1996). Determination of the ^17^O quadrupolar coupling
constant and of the ^13^C chemical shielding tensor anisotropy
of the CO groups of pentane-2, 4-dione and β-diketonate complexes
in solution. NMR relaxation study. Inorg. Chem..

[36] Yang R., Yu J., Liu Y., Wang K. (2005). Effects of inorganic fillers on the
natural photo-oxidation of high-density polyethylene. Polym. Degrad. Stabil..

[37] Nguyen T., Merna J., Kysor E., Kohlmann O., Levin D. B. (2024). Bacterial
degradation of low-density polyethylene preferentially targets the
amorphous regions of the polymer. Polymers.

[38] Zeng S., Lu D., Yang R. (2024). Effects of crystallinity and branched chain on thermal
degradation of polyethylene: A SCC-DFTB molecular dynamics study. Polymers.

[39] Meng X., Jin G., Yang R. (2023). A quantum
chemical and molecular dynamics simulation
study on photo-oxidative aging of polyethylene: Mechanism and differences
between crystalline and amorphous phases. Polym.
Degrad. Stab..

[40] Chamas A., Moon H., Zheng J., Qiu Y., Tabassum T., Jang J. H., Abu-Omar M., Scott S. L., Suh S. (2020). Degradation
rates of plastics in the environment. ACS Sustainable
Chem. Eng..

[41] Boyd R. H. (1985). Relaxation
processes in crystalline polymers: molecular interpretation—a
review. Polymer.

[42] Yao Y., Graf R., Spiess H., Rastogi S. (2009). Influence of crystal
thickness and topological constraints on chain diffusion in linear
polyethylene. Macromol. Rapid Commun..

[43] McCrum, N. G. ; Read, B. E. ; Williams, G. Anelastic and Dielectric Effects in Polymeric Solids; John Wiley & Sons: London, 1967.

