# Supplementary material for: Impact of Carbonyl Group Incorporation in Semicrystalline High-Density Polyethylene
Source: Macromolecules. 2025 Oct 23;58(21):11685–95. doi: 10.1021/acs.macromol.5c02288 (PMC12613794; doi:10.1021/acs.macromol.5c02288)
Supplement: Supplementary file 1 [file ma5c02288_si_001.pdf]

Supporting Information

Impact of Carbonyl Group Incorporation in Semicrystalline High-Density Polyethylene

Afiq Anuar,† Arman Edalat,† Lea Ringelhan,† Qiang Yu,† Maximilian Baur,‡ Albrecht Petzold,† Stefan Mecking,‡ Thomas Thurn-Albrecht,† and Kay Saalwächter*,†

†*Inst. für Physik, Martin-Luther-Univ. Halle-Wittenberg, 06099 Halle (Saale), Germany*

‡*Dept. of Chemistry, University of Konstanz, 78457 Konstanz, Germany*

E-mail: kay.saalwaechter@physik.uni-halle.de

Phone: +49-345-55-28560. Fax: +49-345-55-27161

Contents

Figure S1: SAXS fitting procedure 2

Figure S2: Melt-state SAXS data 3

Figure S3: DSC heating and cooling curves 3

Figure S4: WAXS diffractograms 4

Figure S5: Crystallization kinetics from ^{1}H NMR FID data 4

Figure S6: NMR dipolar second moments (M_2) of all samples 5

Figure S7: ^{13}C CP MAS NMR spectra of ^{13}C-labeled and non-labeled samples . 5

Figure S8: ^{13}C MAS NMR peak deconvolution of the carbonyl region 6

Figure S9: T_1 data of amorphous $CH_{2,a}$ 6

Table S1: Intracrystalline diffusion coefficients (D) and related data 7

Fitting procedure for SAXS Analysis

The small-angle scattering signals were recorded at two sample-to-detector distances 1035.5 mm and 349.9 mm and subsequently combined into a single curve. This combination enables the analysis of scattering intensity over a broad range of the scattering vector s ($s = \frac{2}{\lambda}\sin\theta$), which is essential for further analysis based on the interface distribution function.[1] An example of a SAXS profile of crystallized K1.6-400 and its processing is shown in Figure S1 below. Notably, the SAXS profiles of the KetoPE samples exhibited additional scattering contribution arising from keto group aggregation. While this feature is not easily distinguishable in the crystallized samples, it is more apparent in the melt-state data, as shown in Figure S2. To ensure accurate analysis, this contribution was removed by subtracting the intensity measured in the melt (I_m) from that in the crystalline state (I_sc), yielding a corrected intensity $I = I_\mathrm{sc} - I_\mathrm{m}$. This corrected intensity I was used for all subsequent SAXS analyses.

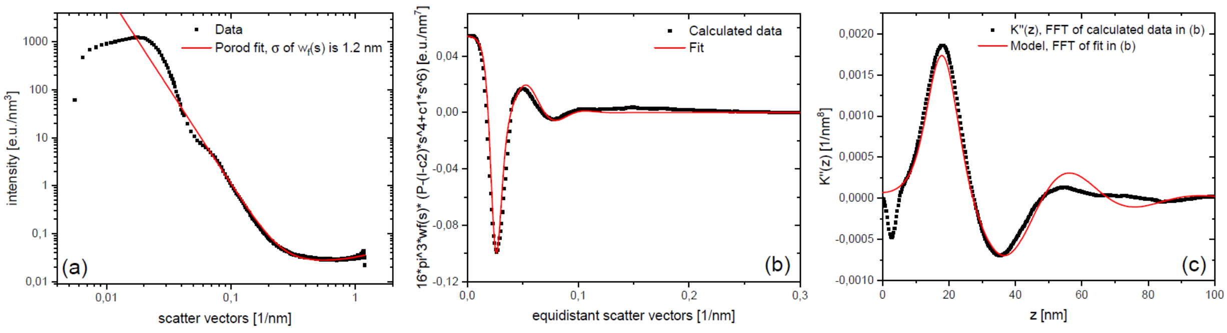

Figure S1: Analysis of SAXS data, illustrated exemplarily on a measurement of K1.6-400 crystallized at 115 °C for 2 hours. (a) The corrected scattering intensity (I) and the corresponding fit consisting of a Porod decay ($P \cdot s^{-4}$), an amorphous halo contribution ($C_1 \cdot s^2$) and a constant contribution from thermal density fluctuation within the amorphous phase (C_2), i.e. $I_\mathrm{Porod} = P \cdot s^{-4} + C_1 \cdot s^2 + C_2$; (b) the function $16\pi^3(P + (C_1 \cdot s^2 + C_2)s^4 - I \cdot s^4) \cdot w_\mathrm{f}(s)$ as calculated from the data in (a) along with a fit based on Fourier transformation of a model interface distribution function $K''(z)$, containing the characteristic structure parameters: d_a and d_c.[1] The purpose of the window function $w_\mathrm{f}(s) = e^{-4\pi s^2 \sigma^2}$, where σ is its width (fixed to 1.2 nm in this study), is used to suppress background noise, which increases in signal at high scattering vectors due to the multiplication by s^4; (c) interface distribution function $K''(z)$ and the Fourier transformation of the fit in (b).

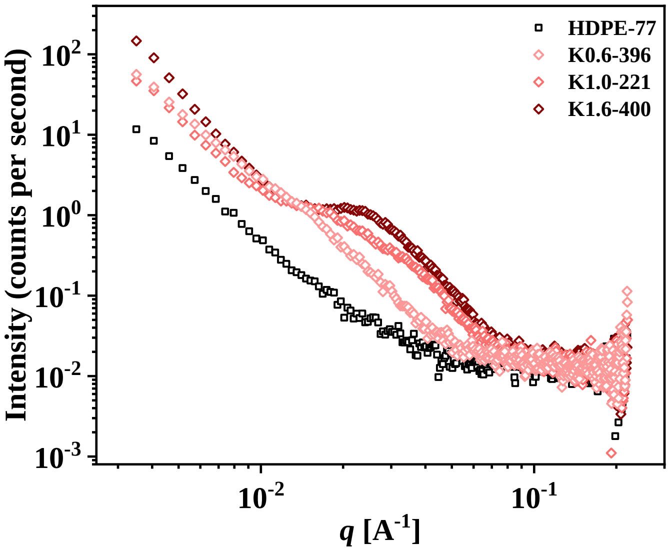

Figure S2: Small-angle X-ray scattering (SAXS) curves of samples HDPE-77, K1.6-400, K1.0-221 and K0.6-396 in the molten state at 180 °C. The KetoPE samples exhibit a distinct scattering peak presumably attributed to the aggregation of keto groups in the melt, which shifts toward higher scattering vectors q ($q = \frac{4\pi}{\lambda}\sin\theta$) with increasing keto concentration. In contrast, the HDPE sample does not show this peak.

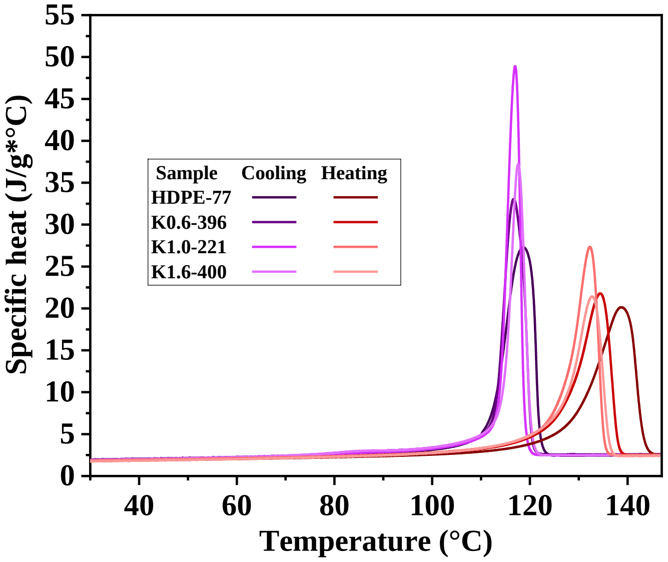

Figure S3: Differential scanning calorimetry (DSC) cooling (purple) and heating (red) scans of HDPE and KetoPE samples at a rate of 10 °C min^{-1} of HDPE and KetoPE samples. Each sample was first molten at 180 °C, then cooled to -60 °C, and subsequently reheated to 180 °C. The plot illustrates the minor shifts in crystallization and melting behavior upon keto incorporation, as recorded during the first cooling/second heating cycle after removal of thermal history at 180°C.

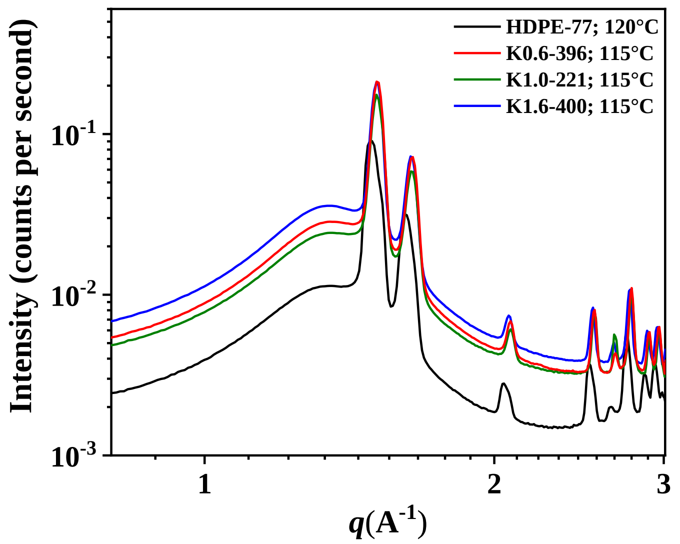

Figure S4: Wide-angle X-ray scattering (WAXS) data of all samples isothermally crystallized for 2 hours at their corresponding temperatures. The scattering peaks of each KetoPE sample overlap with those of HDPE, indicating that the incorporation of keto groups does not change the crystal unit cell of HDPE. The scattering peaks of HDPE are shifted slightly to a lower scattering vector due to larger thermal expansion at the higher crystallization temperature.

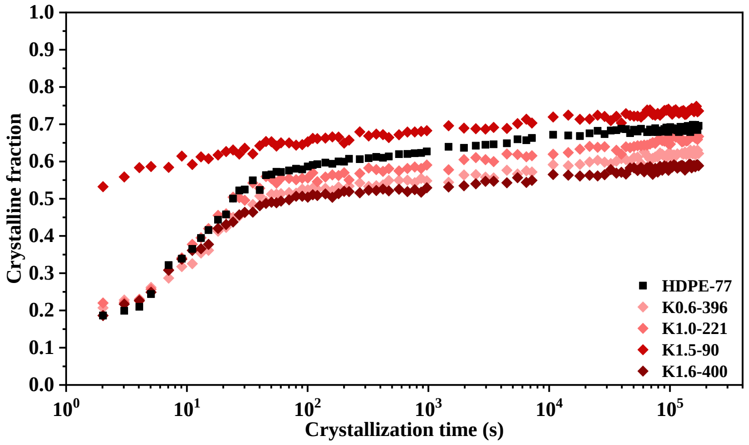

Figure S5: Crystalline fraction from NMR as a function of crystallization time for HDPE and KetoPE samples with varying keto contents, measured at T_c = 124 °C over 2 days from ^{1}H NMR FID analyses. It is seen that all samples exhibit gradual, non-plateau crystallinity development extending beyond the first day, indicating a prolonged secondary crystallization process. This is here attributed to ICD, where ongoing lamellar thickening optimizes the semicrystalline morphology.[1–3] All samples display comparable crystallization rates, suggesting that randomly distributed keto groups have no appreciable impact on the crystallization process. Instead, crystallinity appears to be primarily influenced by M_w rather than keto content, consistent with the observation from the temperature-dependent crystallinity analysis in Figure 2(a). The faster initial crystallization rate observed for K1.5–90 is considered an outlier attributed to temperature instability or nucleating contaminants. Overall, these results indicates that randomly distributed keto groups up to 1.6 mol % exert a negligible effect on the crystallization process. The similarly prolonged secondary crystallization rates across all samples, which associated with ICD, suggest that chain mobility in the crystalline regions remains largely unaffected by the keto inclusion.

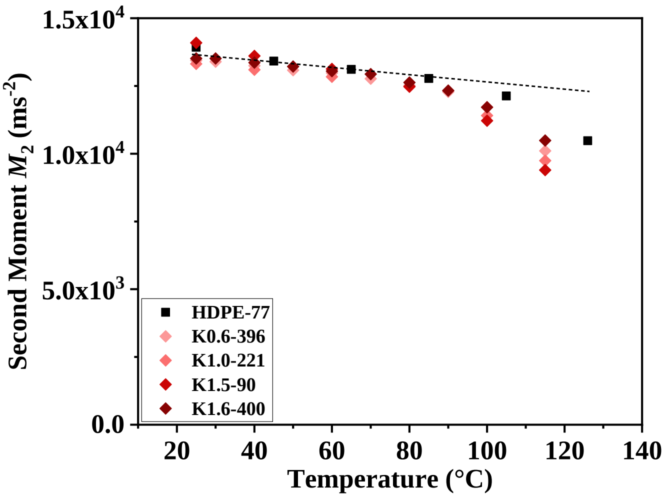

Figure S6: Dipolar second moments (M_2) of all samples extracted from the NMR data in Figure 2(a). All samples show a nonlinear decay with heating at higher temperatures, indicating the presence of ICD.[2,4,5] Moreover, all samples seems to have comparable behaviour suggesting all of them feature nearly identical ICD timescales.

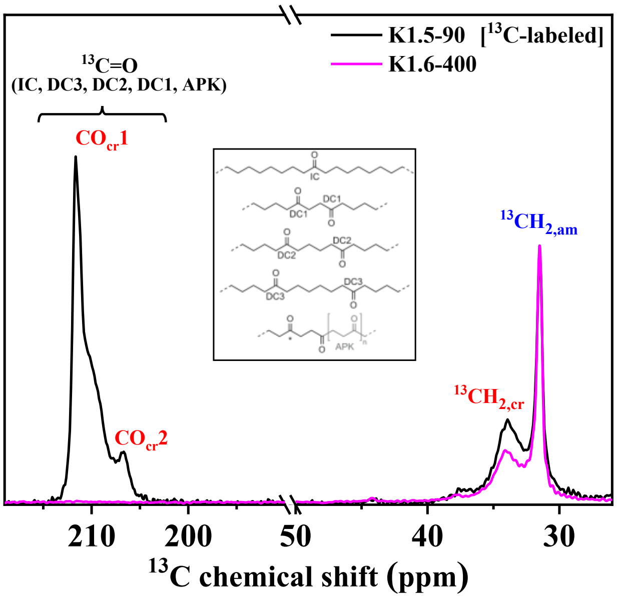

Figure S7: Comparison of ^{13}C cross-polarization (CP) MAS spectra of ^{13}C-labeled K1.5-90 and non-^{13}C-labeled K.16-400 samples at 115°C using a CT of 1.5 ms. The spectra illustrate that, although K1.6-400 has a comparable keto concentration and a much higher M_w than K1.5-90, the low keto group concentration ($\sim$ 1.5 - 1.6 mol %) combined with the 1.1% natural abundance of ^{13}C essentially impedes the detection of ^{13}C resonances from keto groups.

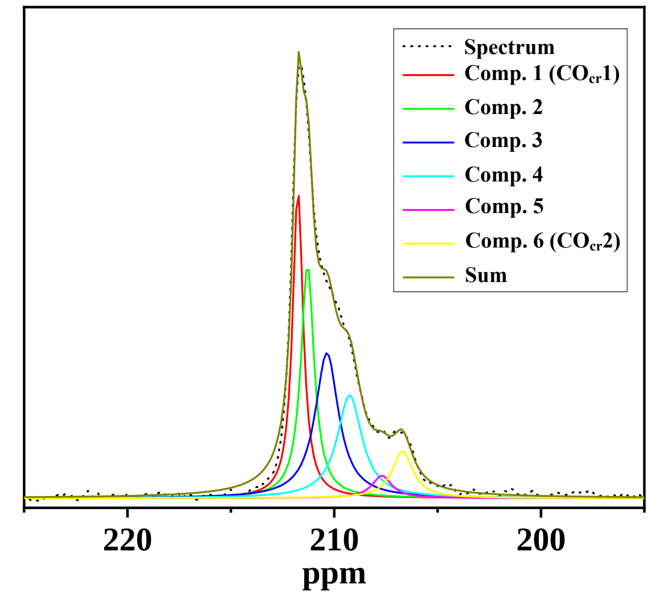

Figure S8: Deconvolution of ^{13}C NMR spectra in the carbonyl resonance region on the example of the K1.5-90 sample at 115 °C (CP with a CT of 1.5 ms, i.e., dominated by immobilized, mostly crystalline groups). Due to spectral overlap and uncertain assignment, only the components 1 and 6 were interpreted as distinct crystal-related CO resonances $CO_{cr}1$ and $CO_{cr}2$ in the main paper.

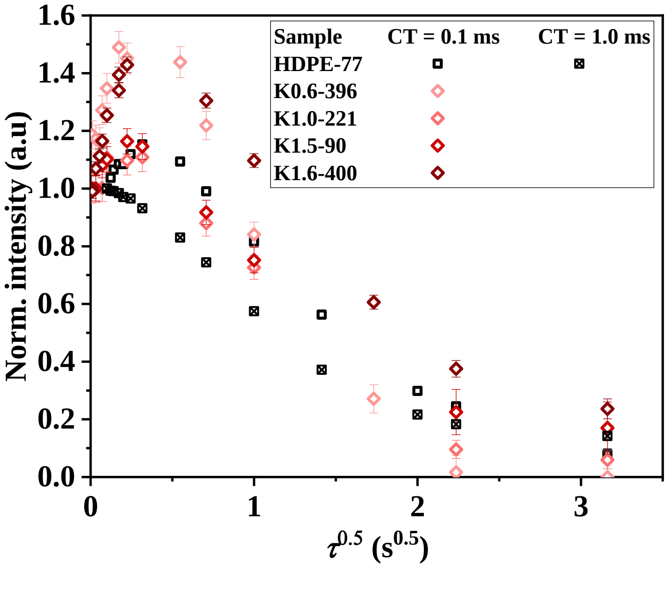

Figure S9: Normalized ^{13}C T_1 relaxation decays of the amorphous $CH_{2,a}$ signal in HDPE and KetoPE samples at 10 kHz MAS and 115°C using two CP contact times (CT = 0.1 ms and 1.0 ms). The buildup observed at short contact time (0.1 ms, open squares) arises from diffusion-mediated magnetization transfer from the highly polarized crystalline phase into the initially less-magnetized amorphous region. At longer contact time (1.0 ms, closed circles), both regions become similarly polarized, eliminating the net transfer and suppressing the buildup. This rules out contributions from NOE, which typically does not diminish at longer contact times. The disappearance of the buildup at 1.0 ms confirms that the observed effect originates from diffusion-mediated transfer, not cross-relaxation, consistent with mechanisms described by Torchia.[6]

Table S1: Summary of logarithmic slopes of the initial decays in ^{13}C T_1 relaxation experiments (after 48 h crystallization, Figure 5a), crystal thickness d_c from SAXS (after 2 h crystallization, see Table III), and intracrystalline diffusion coefficient D for all samples at 115°C (based upon a one-dimensional free-diffusion model, eq. 3). Although the parameters were collected at different crystallization times, the change in crystallinity over this period is modest (typically 3–5%; see Figure S5), suggesting that the slope and d_c values are not significantly affected by the crystallization time difference. D values are comparable across all samples, suggesting that random incorporation of keto groups up to 1.6 mol % neither hinder nor promote intracrystalline dynamics in polyethylene. For the K1.5–90 sample, the D value could not be determined due to the lack of SAXS data, as only NMR analysis was possible with the limited sample amount.

**Sample**	slope ($\times\mathbf{10^{-2}}$)	$\boldsymbol{d_c}$ (nm)	$\boldsymbol{D}$ (nm$^{\mathbf{2}}$/s)
HDPE-77	7.00 ± 0.10	24.24	1.44 ± 0.04
K0.6-396	8.70 ± 0.50	21.91	1.82 ± 0.21
K1.0-221	10.1 ± 0.20	19.37	1.91 ± 0.07
K1.5-90	9.90 ± 0.30	N/A	N/A
K1.6-400	7.80 ± 0.50	20.04	1.22 ± 0.16

References

(1) Schulz, M.; Seidlitz, A.; Kurz, R.; Bärenwald, R.; Petzold, A.; Saalwächter, K.; Thurn-Albrecht, T. The underestimated effect of intracrystalline chain dynamics on the morphology and stability of semicrystalline polymers. *Macromolecules* **2018**, *51*, 8377–8385, DOI: 10.1021/acs.macromol.8b01102.

(2) Anuar, A.; Yu, Q.; Jariyavidyanont, K.; Petzold, A.; Androsch, R.; Thurn-Albrecht, T.; Saalwächter, K. Poly-3-hydroxybutyrate, a Crystal-Mobile Biodegradable Polyester. *Macromolecules* **2024**, *57*, 8507–8518, DOI: 10.1021/acs.macromol.4c00938.

(3) Schulz, M.; Schäfer, M.; Saalwächter, K.; Thurn-Albrecht, T. Competition between crystal growth and intracrystalline chain diffusion determines the lamellar thickness in semicrystalline polymers. *Nat. Commun.* **2022**, *13*, 119, DOI: 10.1038/s41467-021-27752-0 .

(4) Bärenwald, R.; Goerlitz, S.; Godehardt, R.; Osichow, A.; Tong, Q.; Krumova, M.; Mecking, S.; Saalwächter, K. Local flips and chain motion in polyethylene crystallites: a comparison of melt-crystallized samples, reactor powders, and nanocrystals. *Macromolecules* **2014**, *47*, 5163–5173, DOI: 10.1021/ma500691k. Correction: *Macromolecules* **2014**, *47*, 7677–7678, DOI: 10.1021/ma5020963.

(5) Bärenwald, R.; Champouret, Y.; Saalwächter, K.; Schäler, K. Determination of chain flip rates in poly(ethylene) crystallites by solid-state low-field 1H NMR for two different sample morphologies. *The J. Phys. Chem. B* **2012**, *116*, 13089–13097, DOI: 10.1021/jp3061625. Correction: *J. Phys. Chem. B* **2014**, *118*, 12575–12576, DOI: 10.1021/jp51007965.

(6) Torchia, D. A. The measurement of proton-enhanced carbon-13 T_1 values by a method which suppresses artifacts. *J. Magn. Reson. (1969)* **1978**, *30*, 613–616, DOI: 10.1016/0022-2364(78)90288-3.
